# Plague-Contagion in Egypt, and Its Source, with a Supplement on the Quarantine System

**Published:** 1845-01

**Authors:** 


### Art. IV.

1. *Das Pest-Contagium in Egypten und seine Quelle, nebst einem Beitrage zum Absperr-systeme.* Von J. F. R. Grohmann, Med. Dr. des Kaiserthums Oesterreich und Königreichs Sachsen, &c.—Wien, 1844.

*Plague-Contagion in Egypt and its source, with a Supplement on the Quarantine System.* By J. F. R. Grohmann, M.D. of the Empire of Austria and Kingdom of Saxony, &c.—*Vienna*, 1844. 8vo, pp. 258.

*Risposta ai sette Quesiti concernenti la Peste bubonica orientale.* Del Dot. Francesca Grassi, Cavaliere di piu ordini, gia Medico-Chirurgo in capo dello Spedale generale della marina di guerra Egizia, &c. &c.—*Pistoia*, 1843.

*Answer to the Seven Questions concerning the Oriental Bubonic Plague.* By Dr. Francesco Grassi, Knight of several orders, Chief Medical Officer of the General Hospital of the Egyptian Navy, &c.—*Pistoia*, 1843. 8vo, pp. 78.

3. *Correspondence relative to the Contagion of Plague, and the Quarantine Regulations of Foreign Countries*, 1836-43. *Presented to the House of Commons in pursuance of the Addresses of the* 28*th August* 1841, *and* 15*th March* 1842.—*London*, folio, pp. 566.

The appearance of the works just enumerated since the article on the same subject in our Thirty-second Number, has again drawn our attention to the consideration of contagion and quarantine with reference to the propagation of plague. They do not contain much matter of novel interest, yet, as there are several points of importance elucidated by some of the correspondence, and it is stated that the Committee of Privy Council for Trade have at length determined upon opening the question for general consideration, we shall present to our readers the information contained in these works which may be considered as supplementary to our former article.

The work of Dr. Grohmann is written with the view of impressing upon the Austrian government the importance of regulating their quarantine laws upon a secure basis, on the grounds that the plague has a local origin, that it may be circumscribed in its original seat, that the period of quarantine at present pursued is too long, and that the contagious principle may be destroyed by a temperature of 50° to 60° Reaumur. There is nothing new in all this, nor in the reasons which the author gives as his motives for writing. He says he writes, in the first place, because the doctrine of contagion has been lately shaken, particularly by French physicians, whereas their opinions are contradicted by thousands of facts; and secondly, because he has peculiar views upon the epidemic and miasmatic origin of plague, and upon its mode of propagation. He states that he lived for several years and at various periods in the East, partly in Egypt, partly in Macedonia, Thessalia, Wallachia, and some time in Constantinople. During this time, he not only took a most active share in the malignant epidemic which prevailed in Bucharest in 1813, but also met with repeated cases of the sporadic form. The first part of the work consists of an inquiry into the first origin of plague, and whether it be epidemic or miasmatic; in other words, whether it arise from simple meteorological changes in the constitution of the atmosphere or from vegetable or animal emanations, which may be conveyed into the system through its means. The author gives a very fair account of the general causes of disease in Egypt, remarking particularly upon the perpendicularity of the sun's rays, the flux and reflux of the Nile, the emanations of a rich vegetable kingdom, and the South wind. It is interesting and well written, but contains nothing worthy of extract. We have then a section on the climate and condition of Egypt, with regard particularly to the development of plague; but this is so purely speculative as to have little value, the author repeatedly satisfying himself by the employment of certain forms of words which, when analysed, express nothing, where a frank confession of ignorance, or some plain statement of fact, would be far more likely to lead to a knowledge of the truth. He continues his inquiry, first, into the existence of contagion in Egypt, and after roaming through the beaten track of the arguments of the contagionists and their opponents, gives a description of the disease in its different forms, hemorrhagic, petechial, carbuncular, and bubonic, but without throwing any new light upon the subject. He comes to the conclusion that every one else has done, that further inquiry in Egypt is necessary, and passes on to treat of the independence of the origin of the plague of epidemic or miasmatic agency in a long chapter of fifty pages, thoroughly German in the latitude given to theoretical points of dispute, and, to a person acquainted with previous writings on plague, lamentably deficient in novelty or interest. The sum of the whole is that something more than mere epidemic or miasmatic influence is required to produce the specific disease called plague. Another speculative chapter follows on the primary origin of contagion, whether the primordial seeds of contagion are still in operation, or whether new sources are in existence. His conclusion is that the causes are entirely local and closely connected with domestic life and the customs of society. After an appendix in which the author attempts to settle a dispute between Drs. Pezzoni, Oppenheim and Simon, he treats on the means of defence from plague, and in the very commencement of this chapter enters upon a subject which we were the first to open for consideration in our former article—the possibility of a radical extirpation of plague. We shall presently resume this topic. After some remarks on the different means employed in the treatment of plague, Dr. Grohmann enters upon the subject of quarantine regulations. He considers that experience has sufficiently proved their utility in general, but that in detail they are open to serious objections; that while the ingress of plague to healthy countries is sufficiently guarded against, the precautions against its egress from the east are insufficient, but he proposes no means of remedying this defect. He considers the period of quarantine at present pursued too long, but does not define what should be its true limits, although he seems to think five days the extreme period of latency of the disease.

We have gone through this work carefully in the hope of finding some continued passage of sufficient interest for extract, but without success. The book is by no means a bad one, as it gives a very fair account of what is known in the departments of the subject under discussion, but the author does not appear to have clear or defined ideas upon any point, or, to say the least, he fails to convey them to the reader. He constantly enlarges upon purely speculative doctrines, and as often allows their practical bearing to escape him, satisfying himself rather with pointing out errors and abuses, than in devising means for rectifying them. We therefore turn to the Parliamentary Report which consists of correspondence between the Foreign office and our consuls abroad, or with other governments, with regard to the details of quarantine regulations, and a long appendix on the doctrine of contagion. Owing to a proposition which had been made to government with regard to a medical commission in the Levant, to determine the question of contagion, Sir W. Pym was consulted, and he recommended that instead of sending medical men, a circular letter should be addressed to our consuls at Constantinople, Smyrna, Aleppo, &c., requesting them to obtain the opinions of the most eminent medical men in their places of residence. This was accordingly done, and seven questions proposed by Mr. Lewis were sent from the Foreign office as proposed. The following are the seven questions:

1. Is plague communicated by contagion?

2. Is plague communicated by contagion alone, or by other means also; and if so, by what means?

3. Is actual contact with an infected person necessary for communicating plague, or will a close approach to such person communicate the disease?

4. Can substances which have been in contact with an infected person communicate the plague; and if so, what substances?

5. How long may the infection of plague remain dormant in an infected person before it declares itself by evident symptoms?

6. How long will the contagious matter of plague, when lodged in inanimate substances, retain its infecting power?

7. What are the means by which substances containing the contagious matter of plague may be purified?

These questions were submitted to the Royal Society of Medicine in Marseilles, the Board of Health in Alexandria, and to various English, French, and Italian physicians resident in Egypt, Syria, and Turkey. The pamphlet of Dr. Grassi consists of his answers to these questions, with a supplementary address to men of science, entreating them to use their influence in establishing an effectual system first for limiting the extension of the plague, and then to concert measures for its eradication. The answers of Dr. Grassi are exceedingly creditable to him. He has had opportunities superior perhaps to those of any other man for prosecuting his inquiries, and has employed them to the best advantage. He writes in a very clear style, always seizes upon the real point at issue, and bases his arguments upon facts, which are clearly stated and proved by official records to be authentic.

We entered at some length in our former article upon the principal points embraced by the seven questions given above, and our stated opinions are in every respect confirmed by the works at present under notice. We need not therefore recapitulate them, but shall shortly give a few of the most important facts bearing upon the disputed questions. First, the very important evidence of the contagious nature of plague from the results of segregation is furnished by Dr. Grassi. He states, that during the plague in Alexandria in 1834, the squadron of 16,000 persons remained perfectly well, and this not on account of removal from the seat of infection, because other ships in the same situation which had communication with the shore lost many persons. The arsenal, with 6000 labourers, was situated close to the infected quarters, but kept in strict quarantine. During six to seven months, 5 or 6 cases of sudden death occurred, but no decided case of plague. The marine hospital, although surrounded by three villages which were completely deserted from the effects of plague, "remained healthy during the long time that the plague lasted, only because a strict quarantine was maintained within its limits. The contrary took place at the land hospital." (Parliamentary Report, p. 448.) The land hospital was very favorably situated, but the physician was a non-contagionist, did not adopt precautionary measures, and the disease "carried off many victims and subjected the government to great sacrifices." (ib. p. 448.) During this plague the college with its hospital, the populous harem of the viceroy, other inferior establishments, and numerous private families who *shut up*, escaped entirely; while of the Turks, who took no such precautions, it is said, "when the malady declined, more than 100 keys were found at the police office, of houses where the inhabitants had all perished." Dr. Grassi adds, "In the short period of two months I saw fifty-seven dead bodies brought out of the house of a Turk, the treasurer-general of the navy." (ib. p. 448.) Dr. Gaetani Bey gives the numbers of those who were segregated during this pestilence, stating, "that in 32,525 individuals isolated in quarantine, not one case of plague has been proved." (ib. p. 514.) In the answers of Dr. Floquin we find another fact strikingly exemplifying the effects of the segregation of the European population in eastern cities. He says the Turks take no precaution by isolation or quarantine, and "during the plague of 1837, 8 to 10,000 persons died in Smyrna of a population of 40,000 Mussulmen, while scarcely 1000 died of a population of 55,000 Christians." (Report, p. 420.) Dr. M'Carthy states, "I have known an instance of every individual in a harem being carried off successively, although there was not at the time a trace of the disease in the neighbourhood, or in other districts of the capital." (Report, p. 421.) Dr. Grassi gives a table which shows that from 1831 to 1837, "the plague has ten times reached the port of Alexandria from without; that it has been eight times combated and subdued in the Lazaretto; and that in the two instances in which it penetrated into Egypt, the cause was the non-adoption of similar regulations." (Report, p. 461.) Surely further evidence is not required to settle this question.

With regard to communication of plague by *actual contact* alone, we have before expressed our opinion, that it is contrary to reason and experience to suppose that a mere cuticular contact could be dangerous, while the inhalation of the concentrated emanations from a plague patient were innocuous, although it appeared that *close approach* was necessary as a certain dilution of atmospheric air appears to render such emanations harmless. This view is strongly supported by Dr. Laidlaw, who says, "In private houses where ventilation is good, the disease, as a general rule, is confined to one individual alone; wherever a number of persons are assembled in a confined atmosphere, and no attention is paid to ventilation, and the disease appears, every one is cut off." (Report, p. 494.) This is almost the only point at which we are opposed to Dr. Grassi. He supports the doctrine of actual contact, and during plague, in common with other physicians attached to plague hospitals, goes about the city attending other patients, taking the sole precaution of not touching those affected with plague. We are convinced from conversations with several physicians that this freedom of visiting in the cities is one reason which gives a great bias to their minds in support of the belief in contagion by contact alone. If they admitted the possibility of receiving the disease from being in the same room with a patient, it would be necessary that all physicians of plague hospitals should be put in quarantine with their patients. This would be a great inconvenience and pecuniary loss to them, but we maintain that it is a measure absolutely necessary for the public safety, and one which must sooner or later be carried into effect, as it is well known that when plague has been imported into a town, its next attacks are in a great majority of cases in the houses of medical men.

With regard to contagion by clothes or articles of merchandise, Dr. M'Carthy relates a fact which supports the belief that articles of clothing *which have not been exposed to air*, are capable of communicating the plague. "An Armenian banker quitted precipitately his country seat on the Bosphorus, (it was in July) in consequence of an accident of plague among the domestics. Everything was tolerably well purified in the usual manner, with the exception of a couple of trunks, containing fur, shawls and other valuables, in order not to expose them in the absence of the family to the rude manipulations of those rapacious hirelings who gain a livelihood by the luckless service of disinfection. The matter was thus overlooked till the following spring, when the proprietor, wishing again to occupy his summer residence, commissioned some trust-worthy servants to open the trunks with precaution and air the contents; two of the number were attacked and died of plague, though at the same time the public health was unexceptionable." (Report, p. 423.)

We have found no fresh evidence in the works before us showing the length of the period during which plague can remain *latent* in the system, nor anything of value towards determining the time during which inanimate substances retain their infecting power. A commission of Russian physicians has been lately experimenting on this latter question in the Levant. The results of their experiments are not perfectly satisfactory, but they still show that exposure for a short time to a moderate degree of heat, or for a somewhat longer time to a free current of atmospheric air, completely destroys all infecting power, even in the bed-linen of plague patients.

Before leaving this part of the subject, we may mention a remarkable fact which is recorded by Dr. Andreyessky, chief surgeon of the staff at Odessa, showing the possibility of the coexistence of plague with other poisons. He says, "we have seen a pestilential bubo by the side of a venereal bubo, but each occupied the place that was appropriate to it. The two infections had been communicated by a husband to his wife, who, as well as he, had incontestible signs of both diseases. (Report, p. 151.)

We shall now pursue the consideration of quarantine laws. We have spoken of the result of sanitary regulations in countries where the plague only rages after its introduction from other places. Still more important and interesting facts are gathered from observing the effects of those regulations in countries where the plague is supposed to be indigenous. During the last six years, interior quarantines and sanitary police have so far banished plague from Turkey, that the belief has become general in the Levant, that in Egypt alone does the disease originate. Mr. Consul Sandison, writing in 1842, says, "I can recollect no former period during which there has been such a cessation or diminution of the plague throughout the whole extent of the Turkish territory, as since the establishment of quarantines, imperfectly as they have been hitherto regulated." (Report, p. 355.) Experience to the present day has confirmed this; and surely this appears a great boon conferred by the medical profession upon a people, when we remember that in 1812, 3000 daily died in Constantinople alone, and 1500 daily in 1834 and 1836; and in Smyrna between 30,000 and 40,000 (one third of the entire population,) were swept off. Since 1841, very judicious arrangements have been carried on in Alexandria, with the view of separating every case of plague as it arises. (See Parliamentary Report, pp. 401-5.) They met with considerable opposition, but their effects have been hitherto most satisfactory, although some time must elapse before they can be perfectly followed up. One great objection to the establishment of sanitary regulations in Turkey, was the Mahomedan doctrine of predestination. A very interesting dispatch from Lord Ponsonby, inclosing an extract from the Turkish Gazette, of May 1838, shows how the conscientious scruples of these peculiar people were combated. (Report, pp. 276-8.) The arguments are exceedingly well arranged, to the effect that although the accomplishment of the decrees of Providence cannot be prevented by human power, still it is unreasonable not to employ the means which the same Providence has placed in our power for the purpose of self-preservation. As it is necessary that a man eat and drink, to prevent his dying of hunger or thirst, so must he employ remedies to prevent his death from disease. Mahomed himself said, "fly lepers as you would fly from a lion." The plague is a contagious disease, like leprosy, and therefore it is as lawful to flee from one contagion as from another, and take proper precautionary measures, firmly believing at the same time that the plague is only communicated when God permits it. These arguments have been supported by clerical authority, and have done much towards ameliorating the condition of the Turks, sunk as they were under the lethargic influence of an omnipotent fatalism.

This brings us to a question which is daily becoming of greater moment —the possibility of eradicating or totally destroying the plague. We advanced arguments in our last article in support of this belief, from the fact that whole countries formerly subject to yearly visitations of plague have, since the establishment of sanitary laws, been kept entirely free from this scourge. Time has added additional strength to this argument, no new cases of plague having appeared in those countries. Both Dr. Grohmann and Dr. Grassi support this view, though it appears to have escaped the attention of other writers. The former argues that the extirpation of the contagious exanthemata, measles, and scarlatina, and smallpox, notwithstanding the influence of vaccination, is impossible, not only from our ignorance of their original sources, but also from the universality of their extension over the whole globe. But the plague, on the contrary, has a limited seat of origin; it is possible to follow its comparatively limited progress; its native country can be pointed out; even the spot of its generation can be settled without difficulty, and in all probability can be inclosed within very narrow limits. In Dr. Grassi's address to scientific men, at the conclusion of his pamphlet, (pp. 77-8,) he says:

"I also acknowledge the possibility of one day arriving at the point of being able to abolish quarantines and pull down lazarettoes; but not by the means pointed out by the anti-contagionists. If it be desired to attain this end, I will point out the path. The plague being susceptible of being destroyed, and erased from the number of human diseases, it is necessary to aim at this intent (tendere a questo scopo). The plague was found formerly disseminated through all Europe; it was gradually confined to the East, and now its dominion is greatly restricted, because Greece and European Turkey have been liberated. Let the sovereigns of the West, who have such an influence over those of the East, exercise it for this sacred object. Let them assist, and even afford the means which may be necessary. This would be a sacred war, a legitimate intervention, a philanthropic expedition. This would be a great progress for the good of humanity, commerce, and industry. Let it not be believed that this is a work impossssible or difficult. The fanaticism of certain people, who are not yet arrived at the degree of intelligence to be able to discern their own good, would be the chief obstacle. If this, however, could not be overcome by persuasion, it might by force.

"If, on the contrary, we commence where we ought to finish—that is, with a relaxation of precautionary measures, of the prophylactic laws—the oriental plague will shortly introduce itself into Europe, and then all will be lost.

"But who will plead the cause of humanity—make it reach even the throne, and induce the rulers to unite themselves in this sacred alliance? It rests with you, O men of science, who justly enjoy unlimited reputation. You only can be listened to by those who, constituted by Divine Providence heads of kingdoms and monarchies, direct the destinies of the people. Unite yourselves, then, in venerable congress; take into consideration my proposal, dictated by experience and the practice of many years. Paralyse the efforts of innovators, who impudently labour, under the cloak of public good, to recall the already exiled scourge into the breast of Europe; and determine sovereigns to cooperate, so that the eastern monster may be hunted and pursued to extinction in its present insecure retreat." (Risposta, pp. 77-8.)

Allowing for a little Italian imagery and warmth, these are the very arguments advanced by us last year; and we have great pleasure in giving them the support of a physician intimately acquainted, from long residence and official station, with the affairs and conditions of Egypt.

Neither of the writers before us propose any definite measures for obtaining the desired extirpation; we shall therefore offer one or two suggestions. We have found, from repeated and minute inquiries, instituted partly by ourselves, partly at our suggestion through quarantine authorities, that all the sporadic cases of plague occurring in Egypt within the last four or five years, have been observed in certain villages of the Delta. When the plague has been brought into Alexandria, it has always been traced to these villages. It has therefore been an object to determine if these districts were subject to any terrestrial emanations, or if the seeds of contagion were perpetually preserved by any habits or customs of the people. Hitherto we have obtained no conclusive evidence on this question, but there are reasons for inclining to the latter opinion. The inhabitants have a religious or superstitious objection against burying their dead, where they can be reached by water. Now these districts are annually overflowed by the Nile; and the people accordingly preserve their dead above ground, in receptacles which are nothing more or less than a cottage with one door and no windows. In these places the dead are laid, as an Egyptian expressed himself to us, "like herrings in a barrel." Very soon after the death of a person, he is brought with his clothes on, the door is opened, and the corpse laid upon the others, until the place is full. The door is then built up, and another dead-house constructed. The condition of these places varies, according to the time of year and amount of moisture. At times the putrefactive odour is most disgusting; at other times, by the intense heat and dryness of the atmosphere, the bodies were as it were mummied. We have been hitherto unable to prosecute this inquiry further, but hope to be able to do so upon the spot itself, as, *à priori*, we have here a very probable means of keeping up a constant supply of contagion. Clot Bey certainly attaches very little importance to the danger that can arise from the *mephitis* of putrefying corpses. This, however, is not exactly the question. We have seen that they do not always putrefy, and therefore the poison may not be decomposed. And, further, allowing that the dead body some short time after death, may not be capable of propagating contagion, still the clothes and various articles about the person, inclosed as they are soon after death in confined situations, without exposure to air, are just what experience has shown to be active instruments of contagion. We are not inclined to speculate further upon a question which may be settled by the test of experience. If the sporadic causes observed in these villages be traced to contagion from burial-places, means must

LIBRARY OF THE

Orleans Parish Medical Society be taken for destroying these receptacles, and introducing a new mode of sepulture, either by persuasion or force.

It is well known that inoculation with the matter of plague sores has been tried, and with such results as might be expected. It seems also to have been thought that a preventive, as vaccination is of variola, might be discovered. In Dr. Grohmann's work, we find the following quotation from the 'Praxeos Medicæ' of Frank: "Vaccine has been discovered to be a certain antidote against variola—why then cannot an antidote against the plague be found? An epidemic carbuncle is known among the inhabitants of Hungary, and vulgarly called pokolvar. It appears to be the effect of murrain in oxen. Has this analogy with the plague in mankind? Luminosa idea (bright idea!) for if the variola of cows can avert human variola, it is at least worthy of experiment to determine whether the bovine carbuncle transferred to man preserves him from the plague." (Grohmann, p. 212.) We give the quotation as we find it, and, knowing nothing more on the subject, can only hope to see the experiment tried.

Let us now see how the present quarantine laws may be modified upon our *present* knowledge of the natural history of the plague. Our proposition is simply this—do away with quarantine altogether from all places where plague is not actually present, and reduce it to fifteen days for ships coming from parts actually infected at the time of the departure of the vessel. This is almost equivalent to a total abolition of quarantine, as a few Egyptian ports are the only places now affected with any frequency: the united experience of 200 years has proved that it might be done with perfect safety. Why should Morocco and the Barbary States, where plague is never engendered, be debarred from the benefits of free commerce? Algiers enjoys free pratique, and is in free communication with Tunis and Tripoli on the one side, and with Morocco on the other; yet a vessel from either of the latter parts has nine or fifteen days' quarantine, while from Algiers all are in free pratique. Mr. Villiers, our late minister at Madrid, in advocating free communication between Spain and Morocco, says, "By land the States of Morocco have never been invaded by contagion but once, in ancient times. Every description of geographical and physical barrier is opposed to the propagation of disease in that direction; and now that the civilized regulations of the French in Algiers are established between Morocco and the Levant, it is less probable than ever that the public health of this empire (Spain) should be disturbed from that quarter." (Report, p. 206.) Another absurd inconsistency in the present regulations must be noticed. Athens is placed in quarantine as a suspected port; the Ionian Islands are in free pratique, and still have free communication with Athens. So that two ships leave Athens on the same day; one comes direct to Malta or Marseilles, and has seven days' quarantine; the other stops half an hour at Cerigo, sends a boat ashore for a bill of health, and goes to any Mediterranean port without a day's quarantine. We might continue the enumeration of such examples of inconsistent and vexatious impediments to commerce and civilization, and the list might be indefinitely extended if we took up the subject of articles of merchandise, &c. which are considered some as susceptible, and some as non-susceptible of conveying contagion. We shall content ourselves with one example. In company with several naval officers, an officer of the 97th regiment, and another of the artillery stationed at Corfu, we lately made a short excursion in Albania. It was necessary to take a guardiano from the Lazaretto to see that none of us touched any of the Albanians, they being in quarantine. We had a letter of introduction to the Bey of Butrinto, and the old gentleman complimented us by offering his pipe from his own mouth. It had a large amber mouth-piece, and being non-susceptible, it was passed round the whole party with the permission of the guardiano. This fact speaks for itself. The regulations we have just proposed would do away with all such inconsistencies, and establish a uniform and equable system throughout the Mediterranean ports. The experience of 200 years is a guarantee for the security of the measure, supported by all authenticated knowledge of the natural history of plague. How then are we to proceed to secure its adoption? The effect of a conference of European powers appears doubtful. Prince Metternich, who entertains very enlightened views on the subject, endeavoured to obtain a conference of delegates from different governments for the purpose of arranging some uniform system. A great deal of correspondence followed, but we only learn the result incidentally in a letter from our consul at Trieste. He writes that Prince Metternich told him that the project of a congress "went off in consequence of a difference of opinion as to the *place of meeting*." (Report, p. 31.) So much for the etiquette of diplomacy. A committee of medical men, judging from past experience, would probably be productive of no better result. It is painful to see how the conflicting opinions given by medical men on the subject of contagion, often from want of information, perhaps still more often from want of reflection, and occasionally, it is to be feared, from petty professional rivalry, have shaken their credit with legislative persons. Count Molé writes with regard to the meeting of delegates, "There are assuredly points on which we should ultimately agree, without entering into medical discussions, which appear to be scarcely susceptible of solution. Sanitary dispositions have no real sanction but that of experience." (Report, p. 16.)

CHARITY HOSPITAL, —MEDICAL LIBRARY.—

To the press then, as the great instrument for the correction of public abuses, we are obliged to turn, and we have accordingly done the little that lay in our power towards impressing upon those in authority the necessity of basing their regulations upon reason and experience, rather than upon superstition and custom. We have reason to know that our efforts have not been altogether without effect, and it now only remains for a proper system to be authoritatively proposed, to meet with the general consent of the sanitary bodies of the Mediterranean. It is not through the usual diplomatic channels that the object can be obtained. The different boards of health are very little under government control. They enjoy a sort of municipal privilege accorded by general consent of the inhabitants of certain districts, or their representatives, and strengthened by royal charter; and it is through these boards, and not by mere government enactments, that any great or beneficial alteration can be effected in the quarantine laws. We know that the spirit of the whole sanitary body is in favour of a moderate and uniform system, and that the one we have proposed would be supported by the great majority of its members. Its effect would be almost equivalent, as we have shown, to an abolition of quarantine, while the necessity for quarantine officers would still remain. The ships under observation would be much more securely guarded than is at present possible, and the self-interest of lazaretto officers not being interfered with, one great obstacle to former proposed arrangements would be removed.

We confess we should be glad to see this question advocated by Sir William Burnett. His station at the head of the medical department of the navy renders him the natural medical referee of government on all questions connected with our maritime communications; his acknowledged high character and the interest he has already displayed on the subject would combine to ensure a certainty of success to any measure brought forward by him based upon sciencc and truth.

With our present knowledge the necessary means of protection may be reduced to a uniform system, offering few and slight impediments to commerce and civilization, and affording time for prosecuting still further our inquiries into the origin of plague. We may then hope to thoroughly destroy the poison, remove altogether all restrictions to international communication, and emancipate the world from one of its most fearful scourges.

---

